# Noncovalent PAR Binding Guides Proteins to PARP1-Mediated PARylation

**DOI:** 10.1021/acschembio.5c01021

**Published:** 2026-03-13

**Authors:** Arthur Fischbach, Klara Bangert, Alexander Bürkle, Aswin Mangerich

**Affiliations:** † 130368 Max Planck Institute for Biology of Ageing, Molecular Genetics of Ageing Department, 50931 Cologne, Germany; ‡ Molecular Toxicology, Department of Biology, 26567 University of Konstanz, 78457 Konstanz, Germany; § Nutritional Toxicology, Institute of Nutritional Science, 26583 University of Potsdam, 14469 Potsdam, Germany

## Abstract

Poly­(ADP-ribosyl)­ation (PARylation) is a highly dynamic post-translational modification mediated by poly­(ADP-ribose) polymerases (PARPs), influencing DNA damage response, transcription, and cell death. Previously, we showed that noncovalent interactions between PAR and the intrinsically disordered regions (IDPRs) of the p53 C-terminal domain (CTD) mediate the PARP1-dependent covalent modification of its target proteins. In this study, we test whether this mechanism also applies to noncovalent interactions involving the highly basic RGG IDPR of the FUS protein, as well as to domains lacking IDPRs, such as WWE, PBZ, and the macrodomain. We employed a chemical biology approach using a fluorescently labeled NAD^+^ analogue together with fusion constructs that contain defined ADP-ribose-binding domains and validated PARylation acceptors. We find that the p53-CTD, RGG, WWE, and PBZ domains support efficient covalent PARylation, whereas binding through the macrodomain protein Af1521 results in only very weak PARylation. These findings suggest that the mode of PAR binding influences how effectively proteins are directed toward covalent PARylation. This work broadens our molecular understanding of the interplay of noncovalent and covalent PARylation mechanisms and highlights the importance of distinct PAR-binding modes, which may inform future therapeutic strategies aimed at modulating PAR signaling in disease.

## Graphical abstract

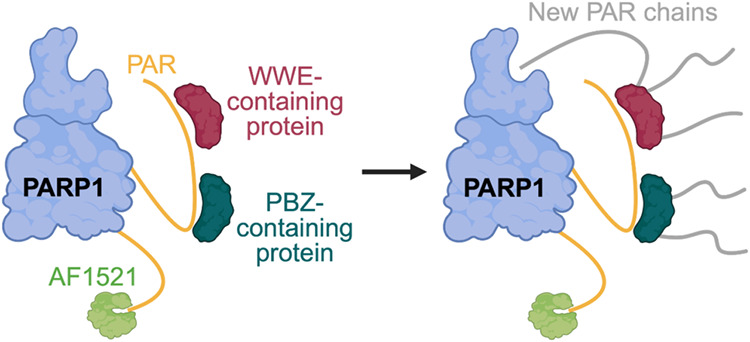

## Introduction

Poly­(ADP-ribosyl)­ation (PARylation) is a post-translational modification catalyzed by poly­(ADP-ribose) polymerases (PARPs), using NAD^+^ as substrate. PARylation plays a crucial role in various cellular processes, including DNA damage response, transcription, and cell death.^1^ Apart from covalent modification of proteins with poly­(ADP-ribose) (PAR), proteins can bind noncovalently to PAR.^2,3^ The recognition and binding of PAR chains are mediated by specific protein domains, including specific intrinsically disordered protein regions (IDPR), WWE, PAR-binding zinc finger (PBZ), and macrodomains^4–7^ (illustrated in Figure 1A). These domains, collectively known as (poly-)­ADP-ribose binding domains (ARBDs), exhibit distinct binding preferences and mechanisms for interacting with PAR chains.^8^ ARBDs can be found in many disease-related proteins, such as FUS (fused in sarcoma), which is an RNA-binding protein and has garnered significant attention due to its involvement in neurodegenerative diseases such as amyotrophic lateral sclerosis (ALS) and frontotemporal lobar degeneration (FTLD). Recent studies have shown that FUS is involved in the DNA damage response mechanism. It can be recruited to sites of DNA damage in a PAR-dependent manner, facilitated by phase separation.^9^ Proteomics studies have identified numerous proteins that undergo covalent PARylation in response to various cellular stimuli.^10,11^ However, the underlying molecular mechanisms that determine which and how proteins are targeted for covalent PARylation remain poorly understood. Our previous work demonstrated that the majority of covalently PARylated proteins contain highly basic, intrinsically disordered protein regions (IDPRs).^12^ We further showed that the tumor suppressor protein p53 contains such a region in its regulatory C-terminal domain (CTD), which facilitates chain-length specific noncovalent PAR binding and targets p53 for covalent PARylation by PARP1.^12–14^

**Figure 1:**
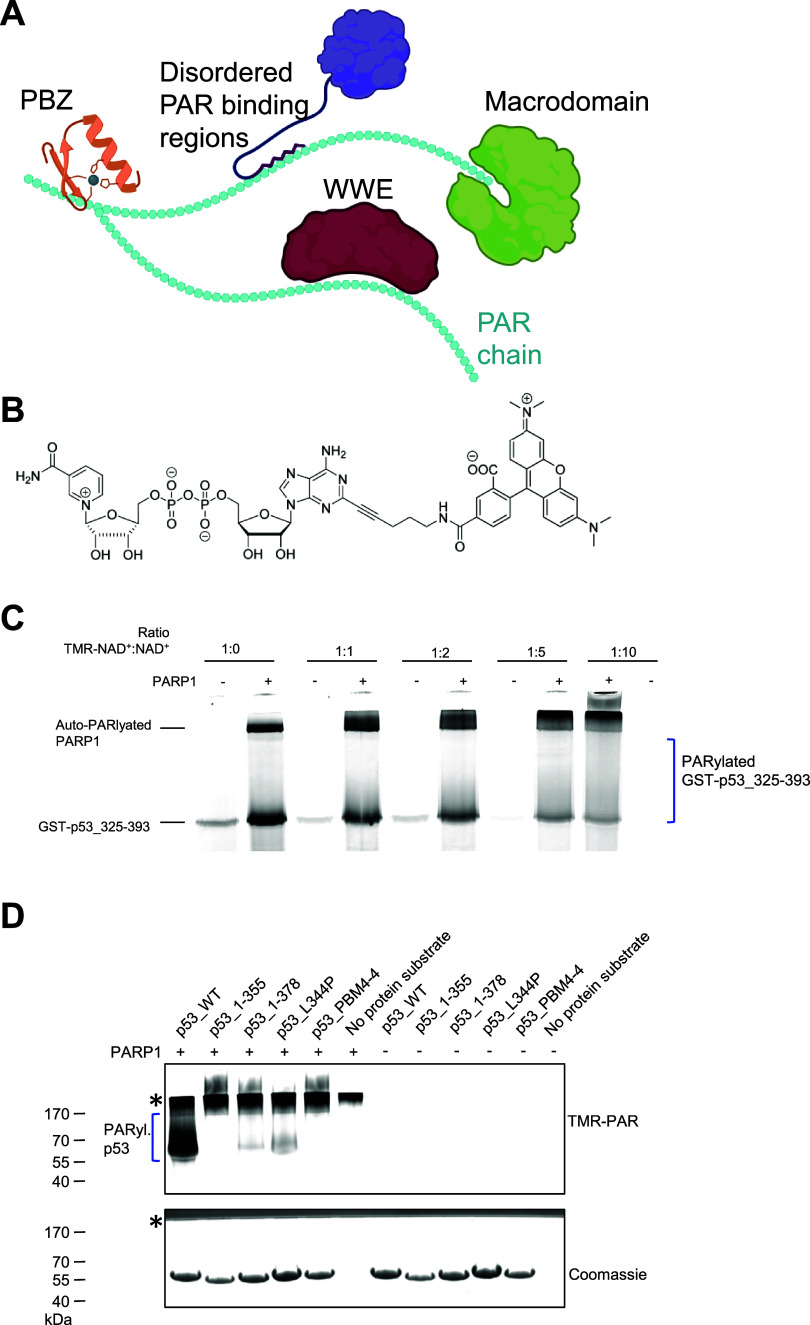
Use of a fluorescently labeled NAD^+^ analogue as reporter for in vitro PARylation assays. (A) Illustration depicting the binding of PAR binding domains/motifs to their respective binding sites on the PAR chain. This includes WWE, PAR-binding zinc finger (PBZ), and macrodomains, as well as disordered PAR binding regions, like from p53. Created with Biorender.com (B) Structure of the fluorescent NAD^+^ analog 2-TMR-NAD^+^. Adapted from Wallrodt et al.^15^ (C) In-gel fluorescence image. Test for different ratios of canonical NAD^+^ to TMR-NAD^+^ using an in vitro PARylation assay. A fluorescent biomolecular imager was used for image acquisition. Total NAD^+^ concentration: 100 μM. PARylation substrate: 5 μM GST-p53_325–393; 150 nM PARP1. (D) In vitro PARylation assay of p53 variants in the presence of 90.9 μM canonical NAD^+^ and 9.1 μM TMR-NAD^+^. Five μM p53 variants and 150 nM PARP1. Coomassie staining is shown as loading control. The asterisk (*) marks the auto-PARylated PARP1 band, which is present in all in vitro PARylation assays. Due to extensive automodification, PARP1 does not migrate from the stacking gel (gray, upper area) to the separation gel section.

The objective of this study was to investigate in greater detail how PARP1 targets proteins for covalent PARylation using a broader spectrum of PAR-binding modules, including domains that lack highly basic IDPRs, such as WWE, PBZ, and macrodomains. We compared these domains in terms of their ability to mediate covalent PARylation, aiming to clarify how different modes of PAR engagement influence this process. To address this question, we employed a chemical biology approach using a fluorescently labeled NAD^+^ analogue together with fusion constructs containing defined ARBDs, enabling direct assessment of PARP1-mediated modification across the different PAR-binding modules tested.

## Results

### Use of a Fluorescently Labeled NAD^+^ Analogue as a Reporter for In Vitro PARylation Assays

We utilized the fluorescently labeled NAD^+^ analogue, 2-tetramethylrhodamin-NAD^+^ (TMR-NAD^+^; Figure 1B), as a reporter for in vitro PARylation assays, which is a method to analyze covalent PARylation of substrate proteins. TMR-NAD^+^ had previously been developed and used by Wallrodt et al.^15–17^ to study in vitro PARylation. It is incorporated into the PAR chain and can be visualized using a fluorescent biomolecular imager. As a reference PAR binding module as well as substrate for PARP1-mediated covalent PARylation, we used the C-terminal domain (CTD) of p53 fused to GST (GST-p53_325‑393_), as previously described and validated.^12^ When TMR-NAD^+^ was used as the sole substrate without canonical NAD^+^ supplementation, a noticeable nonspecific binding to the substrate protein was observed. To minimize such nonspecific binding of TMR-NAD^+^ to the substrate protein, various ratios of TMR-NAD^+^ to canonical NAD^+^ were tested, with the optimal ratio being 1:10 (9.1 μM TMR-NAD^+^ and 90.9 μM canonical NAD^+^), resulting in the lowest nonspecific binding signal (Figure 1C). The in vitro PARylation assay with TMR-NAD^+^ yielded results consistent with our previous findings using p53 variants as substrate and canonical NAD^+^, followed by immunodetection of PAR chains.^12^ Specifically, p53_WT showed covalent PARylation, while PAR-binding-deficient p53_1–355 (referred to here as p53ΔCTD, lacking the p53-CTD) and the PAR-binding-deficient mutant p53_PBM4–4 did not. Tetramerization-deficient p53_L344P and p53_1–378 (containing half of the amino acid sequence of the p53-CTD) exhibited reduced PARylation (Figure 1D). As a control for nonspecific binding, reactions without PARP1 were performed to validate that the fluorescent signal was due to covalent PARylation, and not due to nonspecific binding (Figure 1D).

### The RGG Motif Conveys PAR Binding and PARP1-Mediated Substrate Targeting for Covalent PARylation

The RGG motif of FUS, a highly basic IDPR,^9,12^ was investigated for its ability to convey PAR binding and PARP1-mediated substrate targeting for covalent PARylation. The RGG motif of FUS (amino acid region 468–526, ∼6 kDa), which is known to bind PAR,^9^ was fused to p53ΔCTD (migrates at ∼50 kDa in SDS-PAGE), a p53 truncation deficient in covalent PARylation (Figure 1D).^12^ This variant is referred to here as p53ΔCTD-RGG. A TEV cleavage site was integrated between the two proteins. This fusion protein was subjected to covalent PARylation followed by site-specific protease cleavage. A ∼6 kDa down-shift of the PARylated band confirmed that PARylation occurred in the larger protein fragment, which is p53ΔCTD (Figure 2B). This demonstrates that the RGG motif can convey substrate targeting for covalent PARylation. We assume that RGG is also PARylated, but its PARylation is not visible because the protein is relatively small, comprising only 58 amino acids (approximately 6.4 kDa in total). A PAR overlay assay confirmed noncovalent PAR binding to p53ΔCTD-RGG, whereas p53ΔCTD without the RGG motif showed no noncovalent PAR binding (Figure 2C).

**Figure 2:**
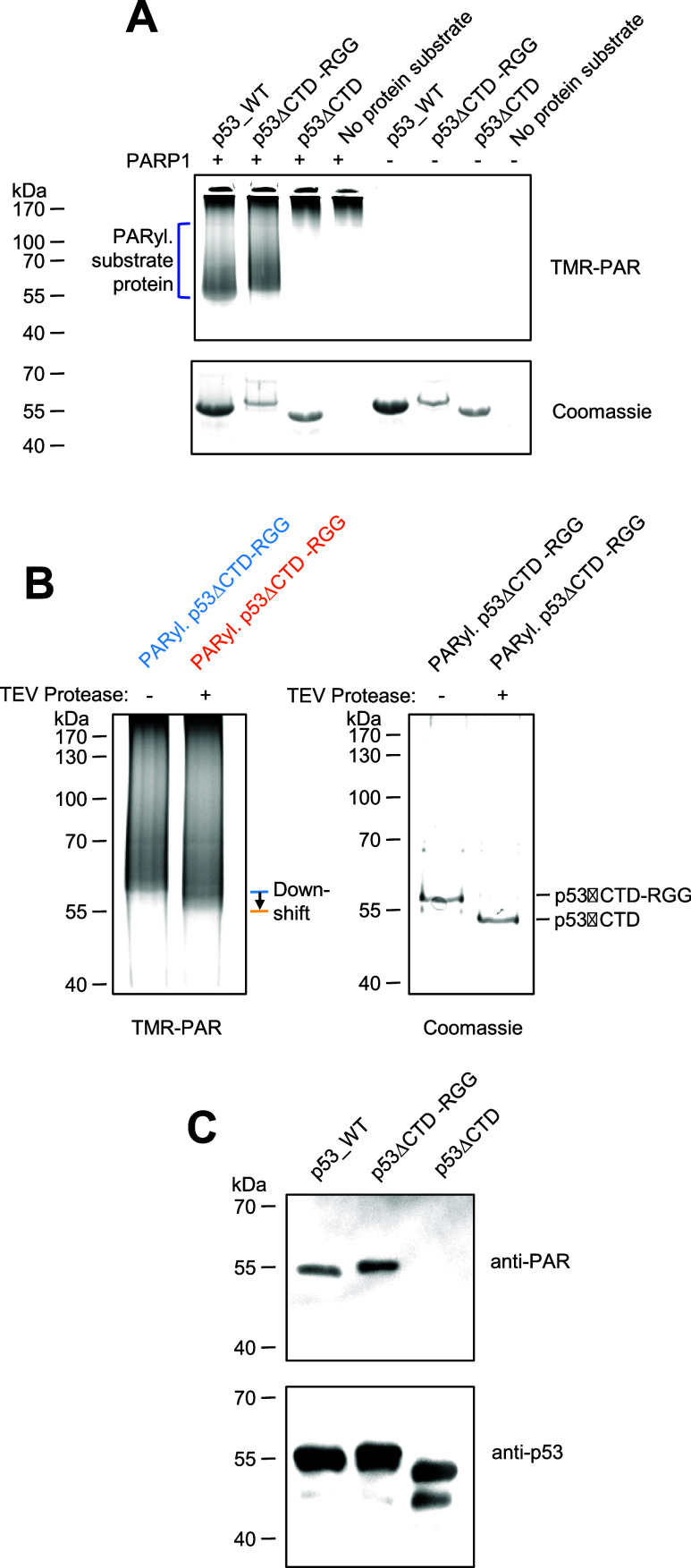
RGG motif, a highly basic IDPR from FUS conveys PAR binding and PARP1-mediated substrate targeting for covalent PARylation. (A) Testing whether p53ΔCTD-RGG (p53_1–355 fused to RGG motif from FUS [FUS_468–526]) is a target for covalent PARylation. TMR-NAD^+^-based in vitro PARylation assay. *N* = 3 independent experiments. (B) TEV cleavage of PARylated p53-RGG. Site-specific TEV cleavage occurs at an engineered cleavage site between the p53ΔCTD and the RGG part. PARylation with 5 μM p53ΔCTD-RGG and 150 nM PARP1. TEV-cleavage with 1.5 μg TEV. *N* = 3 independent experiments. (C) PAR overlay of p53ΔCTD-RGG. Five pmol protein loaded. 15% SDS gel. *N* = 2 independent experiments.

### WWE and PBZ Domains Enable Efficient Covalent PARylation, Whereas the Macrodomain Target Is Only Weakly Modified

We further investigated whether the WWE and PBZ domains, as well as the macrodomain protein Af1521, which all lack IDPRs, can mediate covalent PARylation when incorporated into fusion proteins. Specifically, the WWE domain from human RNF146 (residues 99–183) and the PBZ domain from human APLF (residues 368–451) were examined. APLF was previously reported to be covalently PARylated, which could be abolished by mutating the PBZ domain.^18^ Although previous evidence for covalent PARylation of WWE domain containing proteins is limited, proteomics data reported by Bilan et al. identify the WWE-containing protein TRIP12 as covalently ADP-ribosylated.^19^ Af1521, a protein from the archaeon *Archaeoglobus fulgidus*, consists almost entirely of a macrodomain; therefore, the full-length construct was employed. To the best of our knowledge, the only macrodomain-containing proteins annotated in ADPriboDB as being covalently PARylated are histone variants such as macroH2A, which additionally harbor canonical histone sequences known to support covalent PARylation.^20^ All three domains were fused to GST, which provides additional acceptor sites for covalent PARylation, as previously demonstrated when fused to the p53-CTD (residues 325–393). The GST-p53-CTD fusion protein was therefore used here as a reference control. Although all four constructs bind PAR, efficient covalent PARylation was observed only with the p53-CTD, WWE, and PBZ GST fusions, whereas GST-Af1521 displayed only very weak PARylation (Figure 3A,B) as can be seen by a very faint PARylation band at ∼ 50 kDa. These findings were corroborated using a conventional immunodetection-based PARylation assay using a PAR-specific antibody, which yielded consistent results (Figure 3C,D).

**Figure 3:**
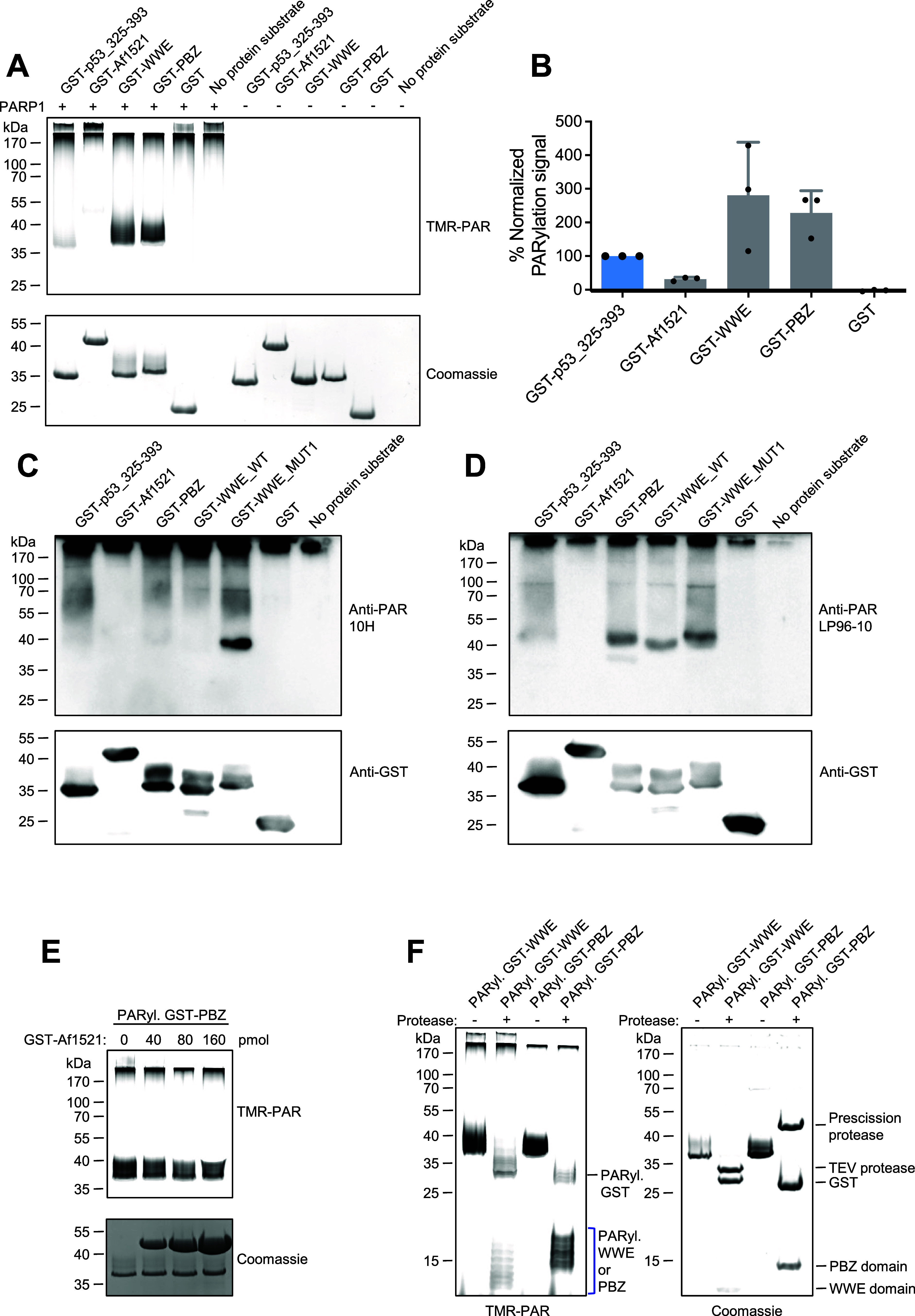
WWE and PBZ domains enable efficient covalent PARylation, whereas the macrodomain target is only weakly modified. (A) TMR-NAD^+^-based in vitro PARylation assay with different PAR binding modules. Five μM protein substrate and 150 nM PARP1. Data are presented as mean values ± SD. (B) Densitometric quantification of the bands shown in (A), with background subtraction based on the signal from PARP1-lacking reactions. *N* = 3 independent experiments. Normalized to GST-p53_325-393. (C) In vitro PARylation assay with only canonical NAD^+^ followed by immunodetection. 2.67 μM substrate proteins. 96 nM PARP1. 100 μM canonical NAD^+^. Detection with mouse monoclonal anti-PAR antibody 10H or (D) with rabbit polyclonal anti-PAR antibody LP96-10. *N* = 1. (E) Test for PAR hydrolysis activity by Af1521. First, the GST-PBZ fusion construct was PARylated (5 μM GST-PBZ and 150 nM PARP1), followed by PARP inhibition and addition of GST-Af1521. Incubated for 20 min at RT. (F) Protease cleavage assay with PARylated WWE or PBZ domain. GST-WWE and GST-PBZ were PARylated, followed by PARP inhibition and site-specific protease cleavage. For the GST-PBZ 1.0 unit of Prescission protease was used and for GST-WWE 0.75 μg of TEV protease.

One potential explanation for the weak PARylation of Af1521 is that the macrodomain may cleave PAR chains. Previous studies reported that Af1521 can hydrolyze protein-mono-ADP-ribose linkages.^21,22^ However, we did not detect any significant PAR cleavage of PARylated GST-PBZ by GST-Af1521 (Figure 3E). Our previous findings showed that the CTD of p53 is sufficient to render a typically nonphysiological substrate like GST susceptible to covalent PARylation by PARP1.^12^ In this study, we explored whether this property extends to the WWE and PBZ domains. To distinguish PARylation events on GST versus the PAR-binding domains, a protease cleavage site (TEV or PreScission) was introduced between GST and the WWE or PBZ domain. Site-specific proteolysis confirmed that PARylation took place on the WWE or PBZ domains as well as on the GST portion (∼26 kDa), as indicated by a ∼10-kDa downshift of the PARylated band as well as the occurrence of the fast-migrating bands of the PARylated WWE or PBZ domains at ∼10–17 kDa or ∼14–19 kDa, respectively (Figure 3F). These results demonstrate that both the PBZ and WWE domains can direct substrate targeting for PARP1-dependent covalent PARylation.

### Noncovalent PAR Binding to Auto-PARylated PARP1 is Responsible for Targeting the GST-PBZ and GST-WWE for Covalent PARylation

Next, we investigated whether the PAR binding ability of PBZ and WWE targets the respective GST fusion constructs for covalent PARylation. To achieve this, we integrated four mutations in the PBZ domain (specifically, four zinc binding cysteines were exchanged to alanines. Referred to here as PBZ_MUT). This approach has previously been shown to abolish PAR binding and covalent PARylation of PBZ-containing proteins, such as APLF and CHFR.^18^ We confirmed that PBZ_MUT lacks covalent PARylation (Figure 4A,C). Moreover, we demonstrated that this mutant no longer binds to auto-PARylated PARP1, as evidenced by Co-IP experiments (Figure 4E).

**Figure 4:**
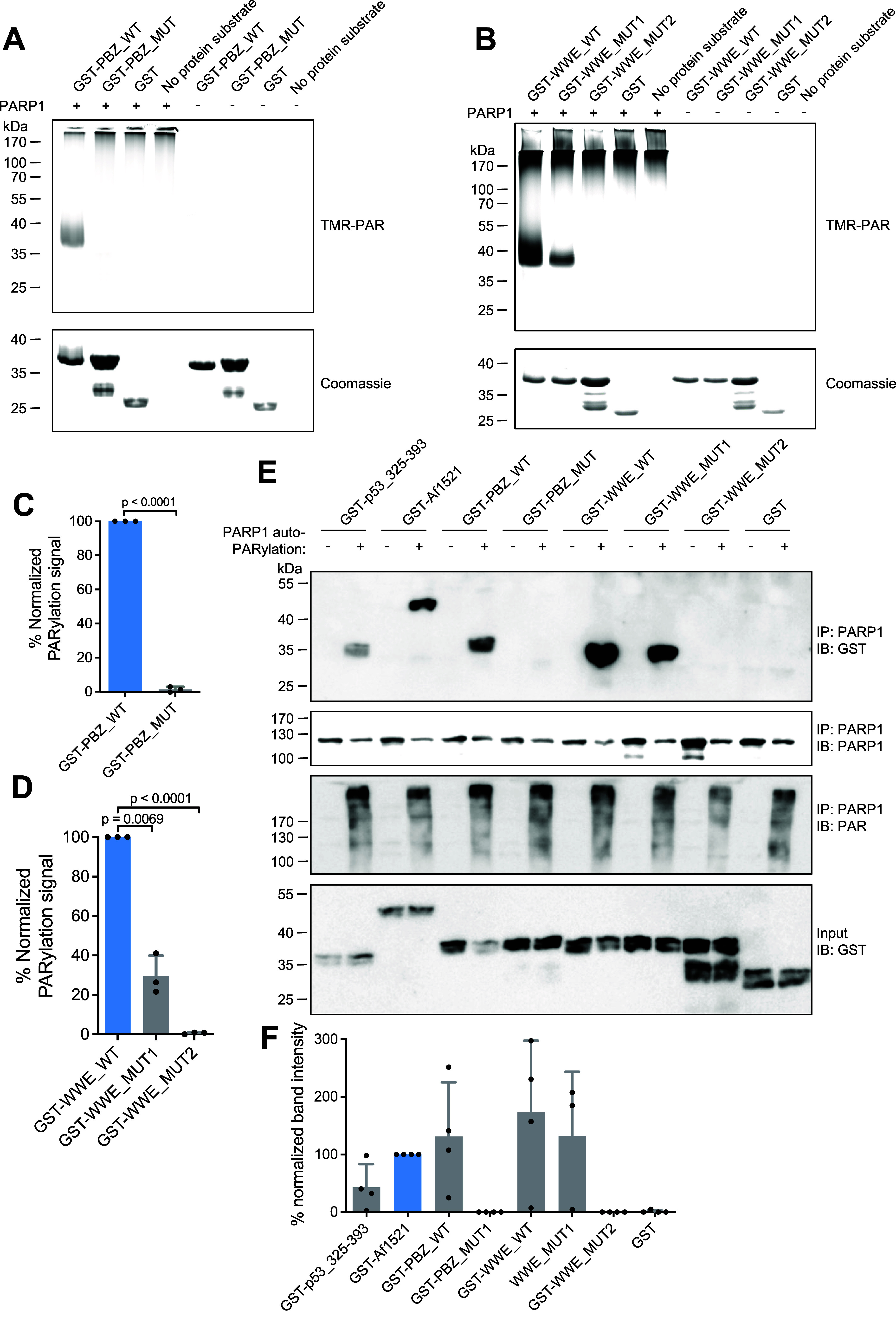
Noncovalent PAR binding to auto-PARylated PARP1 is responsible for targeting the GST-PBZ and GST-WWE for covalent PARylation. (A) In vitro PARylation assay with GST-PBZ_WT and with the PAR-binding-deficient mutant thereof (GST-PBZ_MUT). (B) In vitro PARylation assay with GST-WWE_WT, GST-WWE_MUT1 (mutant with reduced PAR binding) and GST-WWE_MUT2 (mutant with deficient PAR binding). (C) Densitometric quantification of (A) using band signal of reactions without PARP1 for background subtraction. *N* = 3 independent experiments. Data are presented as mean values ± SD. One-sample *t* test. (D) Densitometric quantification of (B) using band signal of PARP1-lacking reactions for background subtraction. Data are presented as mean values ± SD. *N* = 3 independent experiments. One-sample *t* test. (E) Co-immunoprecipitation with auto-PARylated PARP1. Recombinant PARP1 was immobilized on beads and subjected to an auto-PARylation reaction in indicated samples. Recombinant proteins were added and the interaction with PARP1 was analyzed using appropriate antibodies. IP: immuno precipitation, IB: immuno blot. (F) Densitometric quantification of IP: PARP1 IB: GST (top blot) of (E). Normalized to GST-Af1521. *N* = 4.

Additionally, we investigated whether PAR binding is essential for covalent PARylation of the GST-WWE fusion construct. We first introduced the Y107A mutation into the WWE domain (designated GST-WWE_MUT1), which had previously been postulated to abolish noncovalent PAR binding.^7^ Unexpectedly, this mutant still exhibited covalent PARylation (Figure 4B,D). Moreover, we observed that this mutant retained the ability to bind to auto-PARylated PARP1 (Figure 4E), which might be a reason for its continued covalent PARylation. Therefore, we concluded that this mutant was still able to bind PAR noncovalently. To generate a fully PAR-binding deficient mutant, we combined different mutations that had individually been demonstrated to be critical for PAR binding before^7^ (i.e., Y107A, Y144A, R164A) (designated GST-WWE_MUT2), but had never been used in combination. We demonstrate here that this novel mutant is completely deficient in covalent PARylation (Figure 4B,D) and no longer bound to auto-PARylated PARP1 (Figure 4E). Notably, the GST-WWE_MUT1 mutant exhibited reduced PARylation in the in-gel assay (Figure 4B,D), whereas the conventional PARylation assay (Figure 3C) suggested a different outcome. Although this discrepancy cannot be fully resolved, the in-gel approach shown in Figure 4B is generally considered more quantitative than conventional blot-based detection using antibodies, and therefore provides a more reliable assessment in this context. These experiments further indicate that even low-level PAR binding, as observed in WWE_MUT1, is sufficient to target a protein for covalent PARylation by PARP1. Additionally, we observed that the macrodomain-containing protein Af1521 also binds to auto-PARylated PARP1 (Figure 4E) in a Co-IP experiment. As shown above, in this case, we only detected very weak covalent PARylation of the GST-Af1521 fusion protein (Figure 3A,B).

### Search for Putative PAR Binding Proteins Lacking Highly Basic IDPRs in the Proteome of Covalently PARylated Proteins

We previously demonstrated in a bioinformatics approach that the majority of covalently PARylated proteins (68–84%) contain predicted highly basic IDPRs.^12^ However, several PARylated proteins lack these highly basic IDPRs. We therefore analyzed those protein sequences for other potential PAR binding modules. We searched for zinc finger domains, as the zinc finger-containing PBZ domain also exhibits PAR binding ability. Since we observed that low-affinity PAR binding is sufficient for targeting proteins for covalent PARylation, we also searched for other nucleotide binding (ATP, GTP, or NAD^+^) or nucleic acid binding (DNA/RNA) regions.

As a reliable database for covalently PARylated proteins, we utilized the proteomics studies conducted by Zhang et al.^23^ and Martello et al.^10^ These studies analyzed covalently attached PAR residues to amino acids, which is the most accurate method of determining ADP-ribosylated proteins. Our bioinformatics analysis (Figure 5, and Supplementary Data 1) shows that, when highly basic IDPR-containing proteins are excluded, most of the remaining proteins possess zinc finger, nucleotide-binding, or nucleic acid–binding regions. This observation raises the possibility that such regions may also mediate noncovalent PAR binding to automodified PARP1 and subsequent covalent PARylation. Notably, among the set of PARylated proteins without predicted highly basic IDPRs, only 33.3% of the proteins identified by Zhang et al. and 35.9% of those identified by Martello et al. lacked these features (Figure 5). As protein annotations remain incomplete, additional nucleic acid-binding sites may be identified in the future. Together, these findings support the concept that noncovalent PAR binding contributes to the targeting of proteins for covalent PARylation by PARP1.

**Figure 5:**
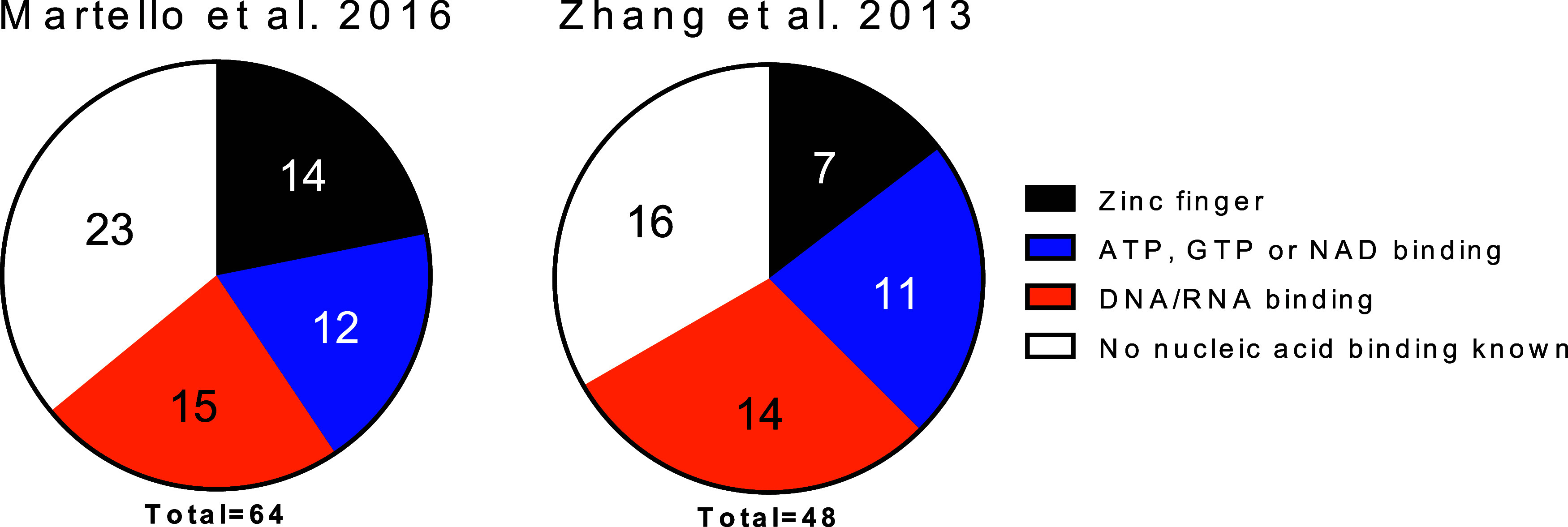
In-silico identification of putative PAR-binding proteins lacking highly basic intrinsically disordered protein regions (IDPRs) within the proteome of covalently PARylated proteins. Previous bioinformatic analyses showed that the majority of PARylated proteins contain highly basic IDPRs; however, a subset of PARylated proteins lacks such regions.^12^ These proteins were analyzed here for other potential PAR binding sites. Specifically, we searched for zinc finger domains, given that the PBZ domain shows also PAR binding. Because a low-affinity PAR binding is sufficient for targeting proteins for covalent PARylation we also searched for other nucleotide binding (ATP, GTP or NAD^+^) or nucleic acid binding (DNA/RNA) regions. The abundance of predicted PAR-binding features in each category is shown in the pie chart.

## Discussion

Our study provides new insights into how PARP1 targets proteins for covalent PARylation, showing that this process is not exclusively dependent on the presence of highly basic IDPRs, such as the p53-CTD (as demonstrated previously^12–14^ ) or the RGG motif of FUS (as shown here). We extend this concept to proteins containing defined ADP-ribose–binding domains, including WWE and PBZ, and show that these domains can also efficiently direct their respective fusion constructs toward covalent PARylation by PARP1. By contrast, the macrodomain protein Af1521 did not substantially mediate PARylation of its GST fusion construct.

These findings raise the hypothesis that the mode of PAR binding may influence how effectively proteins are directed toward covalent PARylation. In particular, our results are consistent with the idea that interaction with internal ADP-ribose units within the PAR polymer is a critical determinant for efficient covalent PARylation. Notably, the domains and motifs that support covalent PARylation, such as IDPRs, WWE, and PBZ, share the ability to bind internal PAR segments, whereas macrodomain-containing proteins are thought to interact primarily with terminal residues,^24,25^ which may account for the limited ability to promote covalent PARylation as observed in this study.

The potential ability of a protein to slide along PAR as a determinant in targeting the protein for covalent PARylation is an intriguing alternative scenario to consider. Previous studies have demonstrated that highly basic IDPRs facilitate sliding along DNA molecules.^26–29^ However, to our knowledge, analogous sliding on PAR chains has not yet been investigated. Exploring this concept may provide valuable insight into how PARP1 identifies and modifies its protein substrates.

Differences in PARylation efficiency might alternatively arise from differences in PAR-binding affinity, with weaker PARylation reflecting weaker binding and thus reduced covalent modification. However, available evidence does not support an affinity-based explanation in the case of the macrodomain. Af1521 binds monomeric ADP-ribose with high affinity in the range of 130 nM to 3 μM^25,30^ and associates with auto-PARylated PARP1 to an extent comparable to WWE and PBZ (Figure 4E), yet is only weakly PARylated in vitro (Figure 3A,B). For comparison, previously reported *K*_d_ values for PAR binding by the WWE domain are 75 ± 2.5 nM (10-mer) and 67 ± 2.3 nM (20-mer) by filter binding and 100 nM by microscale thermophoresis.^31^ For the PBZ domain a *K*_d_ of 0.95 nM was reported using SPR.^32^

These observations indicate that affinity alone cannot account for the inefficient covalent modification of GST-Af1521. Although the molecular basis for the lack of PARylation of the macrodomain construct, despite its high-affinity noncovalent PAR binding, remains unclear, its mode of interaction with PAR may contribute to this effect. Specifically, because the macrodomain binds PAR primarily at its terminus,^24,25^ it may be positioned at a distance from the catalytic center of auto-PARylated PARP1 that is unfavorable for efficient covalent PARylation. This possibility warrants further investigation in future studies.

As a provisional working model, we propose that the sequence of events leading to covalent PARylation of PARP1 target proteins may proceed as follows (Figure 6): (i) PARP1 auto-PARylates itself, generating an elongating PAR chain tethered to its auto-PARylation sites. (ii) As the PAR chain grows, proteins containing PAR-binding modules (such as WWE, PBZ, RGG, or the p53 CTD) can be engaged to internal ADP-ribose units along the polymer. (iii) Internal binding possibly restricts and orients these proteins relative to PARP1, increasing the probability that reactive residues within the substrate become positioned for covalent modification. (iv) Once appropriately oriented, PARP1 transfers ADP-ribose onto the substrate, initiating a new PAR chain on the target protein. In contrast, proteins that predominantly bind to terminal ADP-ribose (e.g., macrodomains) may bind PAR but possibly fail to achieve productive positioning, resulting in weak covalent PARylation. Thus, the mode and position of PAR binding, rather than binding affinity alone, appear to influence whether a protein serves as a substrate for PARP1-mediated covalent PARylation. An important consideration is the possibility of trans-PARylation mediated by other PARP1 molecules. In this context, a PAR-binding protein binds to the auto-PARylated chain generated by one PARP1 molecule, subsequently undergoing covalent PARylation by another PARP1 molecule.

**Figure 6:**
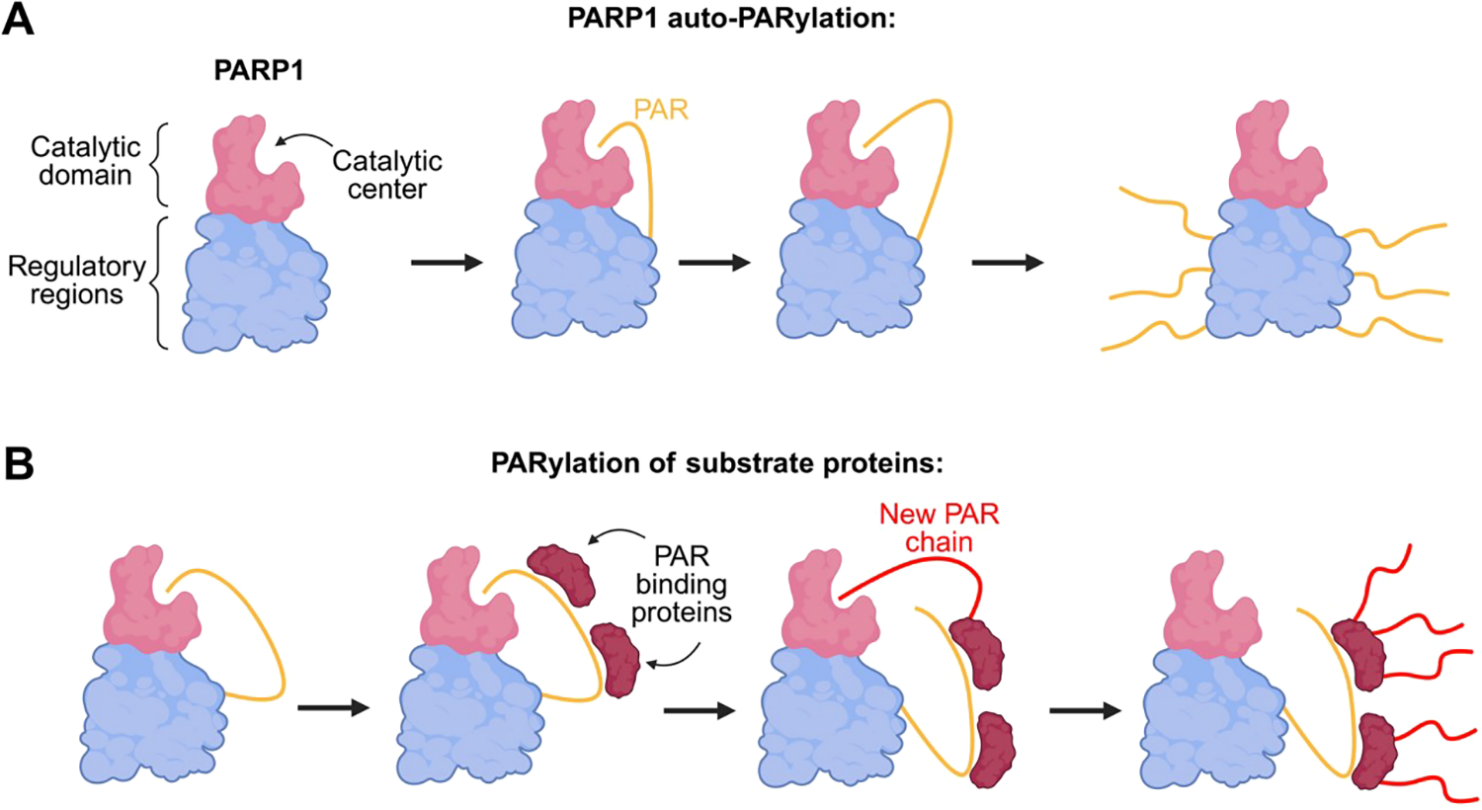
Summary and provisional working model. (A) Simplified hypothetical model for the auto-PARylation of PARP1. Upon activation, PARP1 autonomously PARylates itself. The terminal end of the elongating PAR chain can detach from the catalytic center of PARP1, allowing this step of automodification to be repeated, resulting in the PARP1 molecule being modified by numerous individual PAR chains. (B) Hypothetical model for PARylation of substrate proteins. PAR-binding proteins, such as p53, FUS, PBZ- or WWE-domain proteins, noncovalently bind to the emerging PAR chain from PARP1’s automodification. This elongated PAR chain eventually dissociates from the catalytic center of PARP1. Due to the proximity of the PAR-binding proteins to the catalytic center of PARP1, these PAR-binding proteins are subsequently targeted for the next covalent PARylation. Created with Biorender.com.

In summary, this study demonstrates that covalent PARylation mediated by PARP1 can occur with PAR-binding target proteins possessing a highly basic IDPR (such as the CTD of p53 or the RGG motif of FUS) or a folded PAR-binding module (such as the WWE or PBZ domains), with internal PAR binding appearing to play a key role, whereas terminal PAR binding (as in the case of macrodomains) may substantially reduce modification efficiency. Collectively, these findings provide deeper molecular insight into how PAR-binding proteins are targeted for covalent PARylation by PARP1, highlighting that preceding noncovalent binding to auto-PARylated PARP1 extends beyond highly basic IDPRs to encompass defined ADP-ribose binding domains as well as other potential PAR-interacting regions. This work establishes a foundation for future investigations into the functional consequences of PARylation on diverse protein substrates and may ultimately inform the development of therapeutic strategies aimed at modulating PAR signaling in disease contexts.

## Methods

### Plasmid Construction

All PCR reactions were carried out using a standard 50 μL reaction mix (1 μL KOD Hot Start DNA Polymerase (EMD Millipore), 1× KOD buffer, 1.5 mM MgSO4, 200 μM dNTP mix, 0.3 μM, Fwd/Rvs primer, 200 pg/μL template DNA). DNA was denatured at 95 °C, and extension was carried out at 70 °C. See supporting Table S1 for all primer sequences used in cloning experiments. pET14b::p53_WT, containing His-tagged human p53 was a gift from Martin Scheffner (University of Konstanz, Germany). To generate the truncation variants pET14b::p53_1–324, pET14b::p53_1–355 (p53ΔCDT) or pET14b::p53_1–378, deletion PCRs were performed with pET14b::p53_WT as a template and primers Oligo_01 + Oligo_04, Oligo_02 + Oligo_04 or Oligo_03 + Oligo_04, respectively.

To generate the p53_PBM4-4 mutant pET14b::p53_PBM4–4, the DNA sequence coding for the aa region 357–382 of p53 was deleted by PCR, using the primers Oligo_14 and Oligo_15 and pET14b::p53_WT as template. This PCR product was ligated to annealed double-stranded DNA fragments, encoding the mutated region 357–382 and using Oligo_22 + Oligo_23.

To introduce the L344P aa exchange into p53, site-directed mutagenesis was performed with primers Oligo_56 + Oligo_57, using pET14b::p53_WT as a template

To generate GST-p53_325–393, pGEX2TK::p53_325–393 was received from Martin Scheffner (University of Konstanz).

To generate pET14b::p53ΔCDT-RGG, the RGG motif from the FUS sequence (aa residues 468–526) was extracted by PCR from pEGFP-C1::FUS, which was a gift from Anthony K. L. Leung (Johns Hopkins University School of Medicine, Baltimore, USA), using primers Oligo_01 and Oligo_02. From pET14b::p53_WT, aa region 356–393 of p53 was deleted by PCR using primers Oligo_03 and Oligo_04. Both PCR products were ligated.

pGEX4T1::WWE_WT as well as the Y107A mutant (WWE_MUT1; pGEX4T1::WWE_Y107A) were gifts from Wenqing Xu (University of Washington, Seatlle, USA) and contain the WWE domain from human RNF146 (aa residues 99–183).

To generate the Y107,144A,R164A mutant of the WWE domain (WWE_MUT2; pGEX6P1::WWE_ Y107,144A,R164A), site-directed mutagenesis was performed with pGEX4T1::WWE_Y107A as template and using primers Oligo_05 and Oligo_06, as well as Oligo_07 and Oligo_08 in a second round of mutagenesis.

pGEX6P1::PBZ_WT was a gift from David Neuhaus (MRC Laboratory of Molecular Biology, Cambridge, U.K.) and contains the PBZ domain from human APLF (aa residues 368–451). To generate the C379,385,421,427A mutant of the PBZ domain (PBZ_MUT; pGEX6P1::PBZ_ C379,385,421,427A), site-directed mutagenesis was performed using primers Oligo_09 and Oligo10, as well as Oligo_11 and Oligo_12 in a second round of mutagenesis.

All steps during molecular cloning were controlled by analytical restriction enzyme digestion and by Sanger sequencing.

### Protein Purification

Human p53, mutants and truncation variants thereof as well as p53ΔCDT-RGG (p53_1–355 + FUS_468–526) were expressed in *Escherichia coli* BL21­(DE3). Expression was induced at an OD600 of 0.6–0.8 with 20 μM IPTG, followed by an incubation for 12 h at 16 °C and 4 h at 10 °C. After centrifugation, cells were snap-frozen in liquid nitrogen. Cells were thawed and resuspended in lysis buffer [50 mM sodium phosphate pH 8.0, 300 mM NaCl, 10 mM 2-mercaptoethanol, 10 mM imidazole, 1 mg mL^–1^ lysozyme, 1× EDTA-free protease inhibitor cocktail (Roche)]. Cells were sonicated, followed by DNA digestion with 5 μg/mL DNase I for 30 min. The insoluble fraction was removed by centrifugation at 15,000 *g* for 30 min. The soluble fraction was filtered and loaded on an ÄKTA chromatography system (GE Healthcare), using a 1-ml HisTrap column (GE Healthcare). Elution was performed with a linear gradient from 10 to 500 mM imidazole in a buffer, consisting of 50 mM sodium phosphate pH 8.0, 300 mM NaCl. Elution fractions were dialyzed and thrombin-cleavage was performed overnight for His-tag removal in a buffer, consisting of 20 mM sodium phosphate pH 8, 150 mM NaCl, 10 mM 2-mercaptoethanol. Thereafter, thrombin was inhibited with 0.1 mg mL^–1^ Pefabloc (Roche). A second purification step was performed with a HiTrap heparin HP 1-ml column (GE Healthcare), using a linear gradient from 150 to 1000 mM NaCl in a buffer, consisting of 20 mM sodium phosphate pH 8.0.

GST-p53_325–393 and GST were expressed and lyzed as described above, with a modified lysis buffer (50 mM sodium phosphate pH 7.0, 300 mM NaCl, 5 mM 2-mercaptoethanol, 1 mg mL^–1^ lysozmye, 1× protease inhibitor cocktail (Roche)). GST-WWE_WT (human RNF146 aa 99–183) and the Y107A mutant were expressed as described previously.^5,7^ The Y107,144A,R164A mutant was expressed as described for p53. Cell lysis occurred in the modified lysis buffer. GST-PBZ_WT (human APLF aa 368–451) and the mutant GST-PBZ_MUT were expressed as described previously.^4^ GST-PBZ_WT was lyzed in a buffer consisting of 50 mM sodium phosphate pH 7.5, 1 M NaCl, 150 μM ZnSO_4_, 5 mM 2-mercaptoethanol, 1 mg mL^–1^ lysozmye, 1× EDTA-free protease inhibitor cocktail (Roche). GST-PBZ_MUT was lyzed in a buffer consisting of 50 mM sodium phosphate pH 7.5, 300 mM NaCl, 5 mM 2-mercaptoethanol, 1 mg mL^–1^ lysozmye, 1× protease inhibitor cocktail (Roche). To the cleared lysate 0.6 mL of glutathione sepharose 4B (GE Healthcare) was added and incubated rotating for 90 min at 4 °C. Beads were washed with 15 mL GST wash buffer (50 mM sodium phosphate pH 7.0, 300 mM NaCl) for 10 min, while rotating. After centrifugation and removal of the supernatant, 7.5 mL GST wash buffer were added to the beads and the slurry was poured into an empty gravity flow column. The column was washed with 5 mL of GST wash buffer. Elution was performed with 14 mL of 10 mM glutathione in GST wash buffer. GST-Af1521 was expressed and purified according to a previous study.^10^ The used plasmid DNA pGEX-4T1::Af1521 was received from Michael L. Nielsen (University of Copenhagen, Denmark). Human PARP1 was expressed in *Sf9* cells and purified as described previously.^33^

### In-Vitro Coimmunoprecipitation (Co-IP)

The in vitro Co-IP was performed as previously described.^12^ Briefly, recombinant human PARP1 (1 μg) was incubated with 10 μg anti-PARP1 antibody (FI-23)^35^ for 1 h at 4 °C in 300 μL IP buffer (50 mM Tris-HCl pH 7.4, 150 mM NaCl, 1% NP-40, 0.5% deoxycholate). 10 μL Protein G sepharose beads (Sigma-Aldrich) were added and incubated for 2 h at 4 °C, while rotating. Indicated samples were subjected to auto-PARylation of PARP1 by adding 10 mM MgCl_2_, 7.7 μg/mL self-annealed oligonucleotide GGAATTCC, 1 mM DTT and 100 μM NAD^+^. Samples were incubated for 5 min at 4 °C, while rotating. After centrifugation at 2400 *g* for 20 s, beads were washed with 500 μL wash buffer (50 mM Tris-HCl pH 7.4, 150 mM NaCl, 0.1% NP-40, 0.05% deoxycholate), followed by a second washing step, using 250 μL IP buffer. After centrifugation and removal of the supernatant, 300 μL IP buffer were added, together with 1 mM DTT and 50 pmol of rec. GST-coupled proteins. Incubation took place for 1 h at 4 °C while rotating. Supernatant was removed and 30 μL of the supernatant was used as input. Beads were washed twice for 5 min in 500 μL IP buffer and once for 5 min in 500 μL wash buffer while rotating. Proteins were eluted from beads by adding 22 μL of 2× SDS sample buffer, incubating 5 min at 95 °C and subjected to SDS-PAGE followed by Western blotting. The rabbit polyclonal anti-PARP1 antibody H250 (Santa Cruz) and the rabbit polyclonal anti-GST antibody A6 (Santa Cruz) were used for immunodetection.

### In-Vitro PARylation Assay

Five μM rec. substrate protein was PARylated in PBS buffer, in the presence of 10 mM MgCl_2_, 7.7 μg/mL self-annealed oligonucleotide GGAATTCC, 1 mM DTT, 150 nM rec. PARP1, 90.9 μM canonical NAD^+^ and 9.1 μM TMR-NAD^+^ in a final volume of 6.6 μL for 20 min at RT. Proteins were separated by 15% SDS-PAGE, followed by fluorescence detection with a Typhoon FLA 9500 biomolecular imager (GE Healthcare) using the Cy3 filter. To visualize the prestained protein marker (PageRuler, Thermofisher Scientific), the Cy5 filter was applied. Protein loading was controlled by Coomassie staining (PageBlue, Thermofisher Scientific). Each in vitro PARylation experiment was performed at least in three independent experiments.

### Site-Specific Protease Cleavage Assay

Rec. protein was subjected to an in vitro PARylation using TMR-NAD^+^, as described above. however, in a final volume of 10 μL. After 20 min at RT, PARylation was stopped by addition of 10 μM ABT-888 (Selleckchem). To the PARylation of GST-PBZ_WT one unit of Prescission protease (GE Healthcare) was added and 0.75 μg of TEV protease for GST-WWE_WT. Samples were incubated for 18 h at 4 °C. Protease cleavage was stopped by addition of SDS sample buffer and samples were subjected to SDS-PAGE, using a 15% acrylamide gel, followed by fluorescence detection with a Typhoon FLA 9500 biomolecular imager (GE Healthcare) using the Cy3 filter. To visualize the prestained protein marker, the Cy5 filter was applied. Protein loading was controlled by Coomassie staining. Each protease cleavage assay was performed in three independent experiments.

### PAR Overlay Assay

Five pmol proteins were blotted on a nitrocellulose membrane (GE Healthcare) either by slot-blotting or by SDS-PAGE and semidry blotting, respectively. The membrane was incubated in a solution containing 0.2 μM PAR (synthesized and purified as described previously)^33^ in TBST buffer (150 mM NaCl, 10 mM Tris pH 8, 0.05% Tween 20) for 1 h at RT, followed by three washes of 10 min in TBST buffer, containing 1 M NaCl. PAR concentrations refer always to ADP-ribose moieties. The membrane was blocked in TBSMT (TBST with 5% skimmed milk) for 1 h and subsequently PAR was detected using the mouse monoclonal 10H anti-PAR antibody and a HRP-coupled secondary antibody (Dako). Detection of p53 was performed with the mouse monoclonal DO-1 antibody (Merck). As loading control for slot-blotting, SYPRO ruby staining (Thermofisher Scientific) was used directly after blotting. Each PAR overlay experiment was performed at least in 3 independent experiments.

## Supplementary Material





## Data Availability

All data supporting the conclusions of this study are included in the paper and/or the Supporting Information. Source data are provided with this paper.

## Notes

Uncropped images of gels and Western blots; primer sequence information in Table S1 (PDF)
Bioinformatics search for proteins possessing zinc finger, nucleotide-binding, or nucleic acid–binding regions in proteomic data sets of covalent protein PARylation (XLSX)
