# Supplementary material for: Noncovalent PAR Binding Guides Proteins to PARP1-Mediated PARylation
Source: ACS Chem Biol. 2026 Mar 13;21(4):779–89. doi: 10.1021/acschembio.5c01021 (PMC13097088; doi:10.1021/acschembio.5c01021)
Supplement: Supplementary file 1 [file cb5c01021_si_001.pdf]

Supporting Information for:

Non-Covalent PAR Binding Guides Proteins to PARP1-Mediated PARylation

Arthur Fischbach[1,2]*, Klara Bangert[2], Alexander Bürkle[2] and Aswin Mangerich[3,2]*

[1]Max Planck Institute for Biology of Ageing, Molecular Genetics of Ageing Department, 50931 Cologne, Germany

[2]Molecular Toxicology, Department of Biology, University of Konstanz, 78457 Konstanz, Germany

[3]Nutritional Toxicology, Institute of Nutritional Science, University of Potsdam, 14469 Potsdam, Germany

* Corresponding authors

To whom correspondence should be addressed. Tel: +49-(0)331 977-295515; Email: mangerich@uni-potsdam.de

Correspondence may also be addressed to Arthur Fischbach. Email: afischbach@age.mpg.de

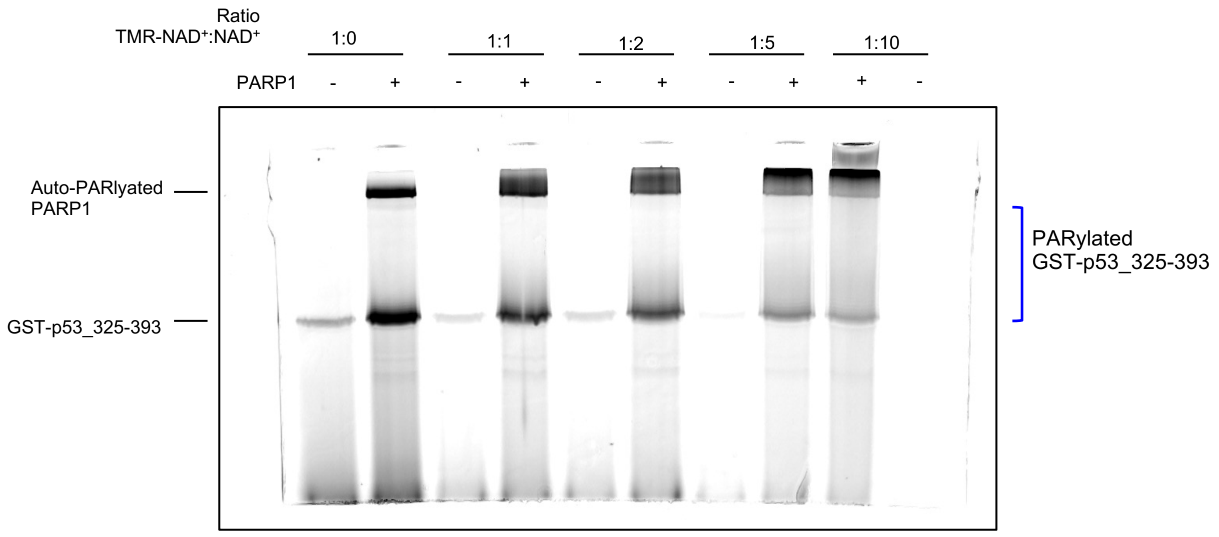

Figure S1. Uncropped gel image from Figure 1C.

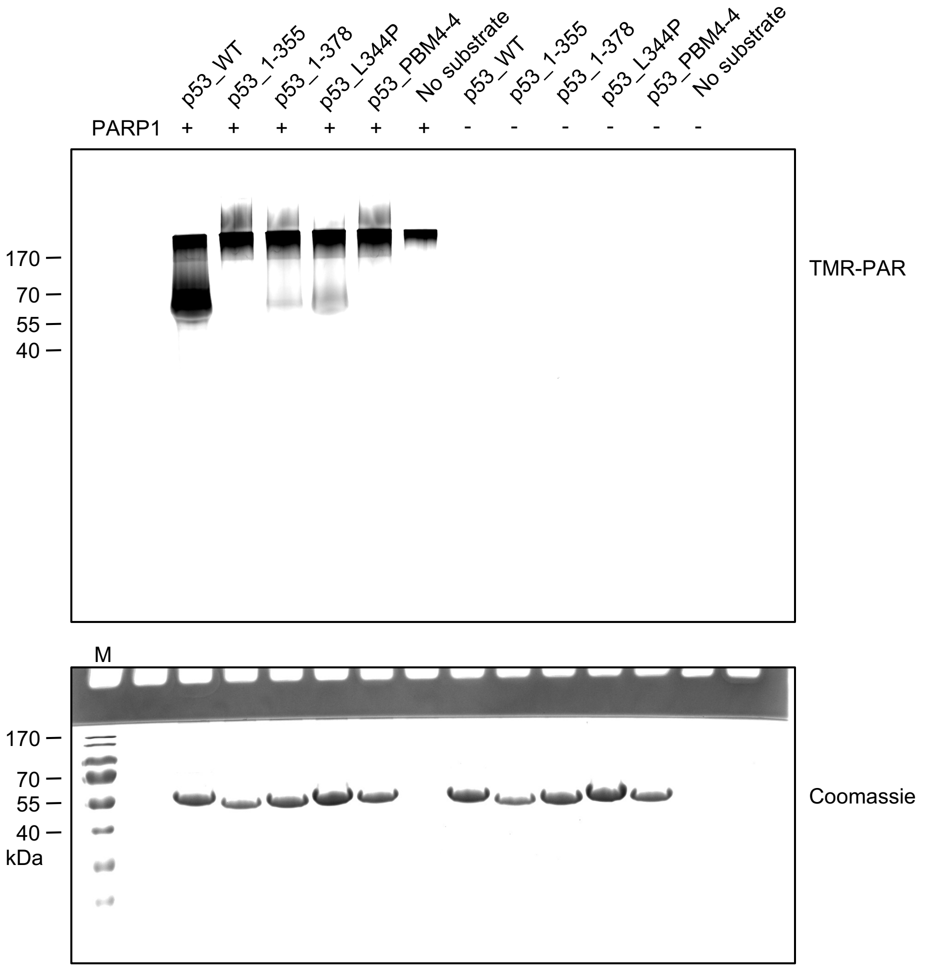

Figure S2. Uncropped gel images from Figure 1D. M: Molecular weight marker

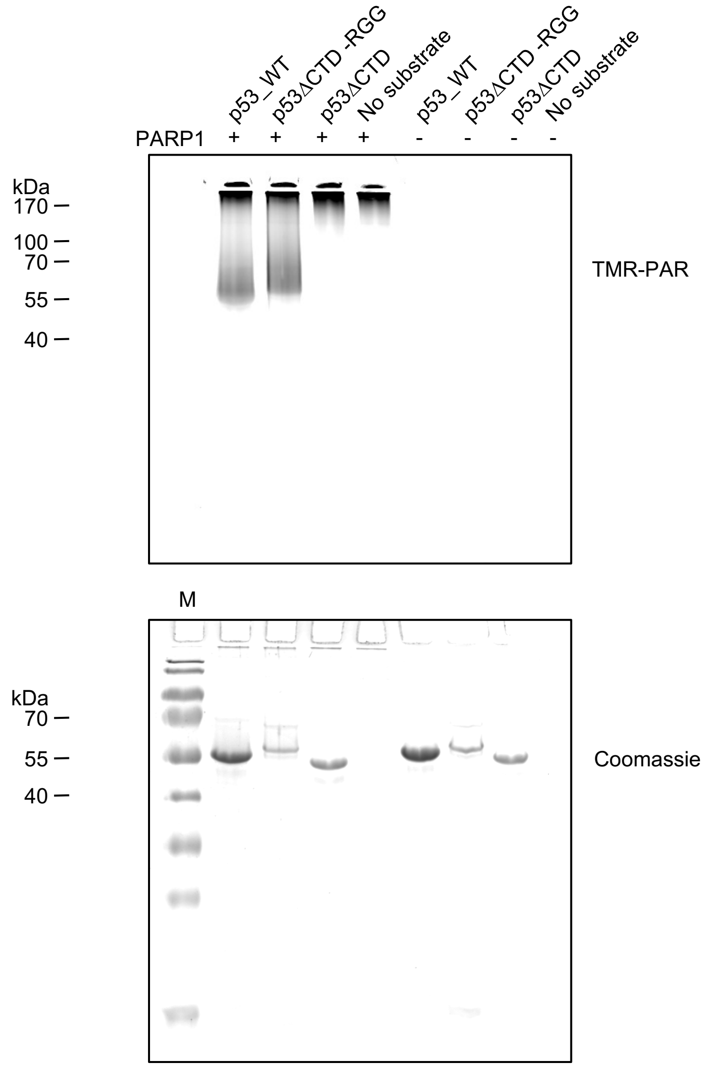

Figure S3. Uncropped gel images from Figure 2A. M: Molecular weight marker

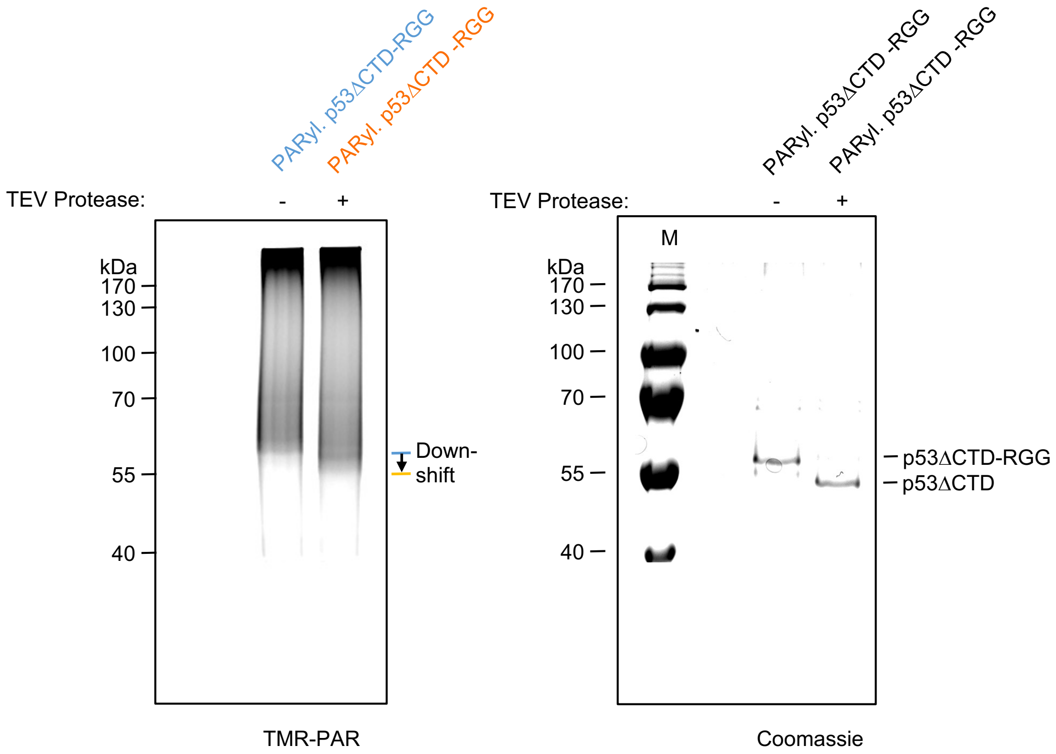

Figure S4. Uncropped gel images from Figure 2B.
M: Molecular weight marker

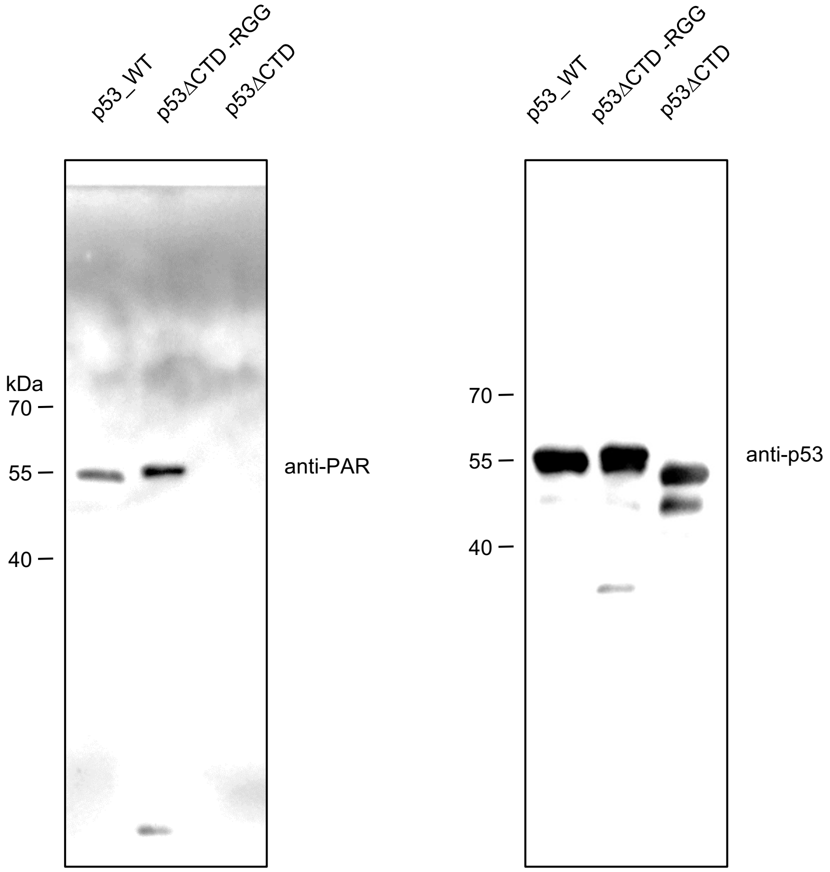

Figure S5. Uncropped Western blots from Figure 2C.

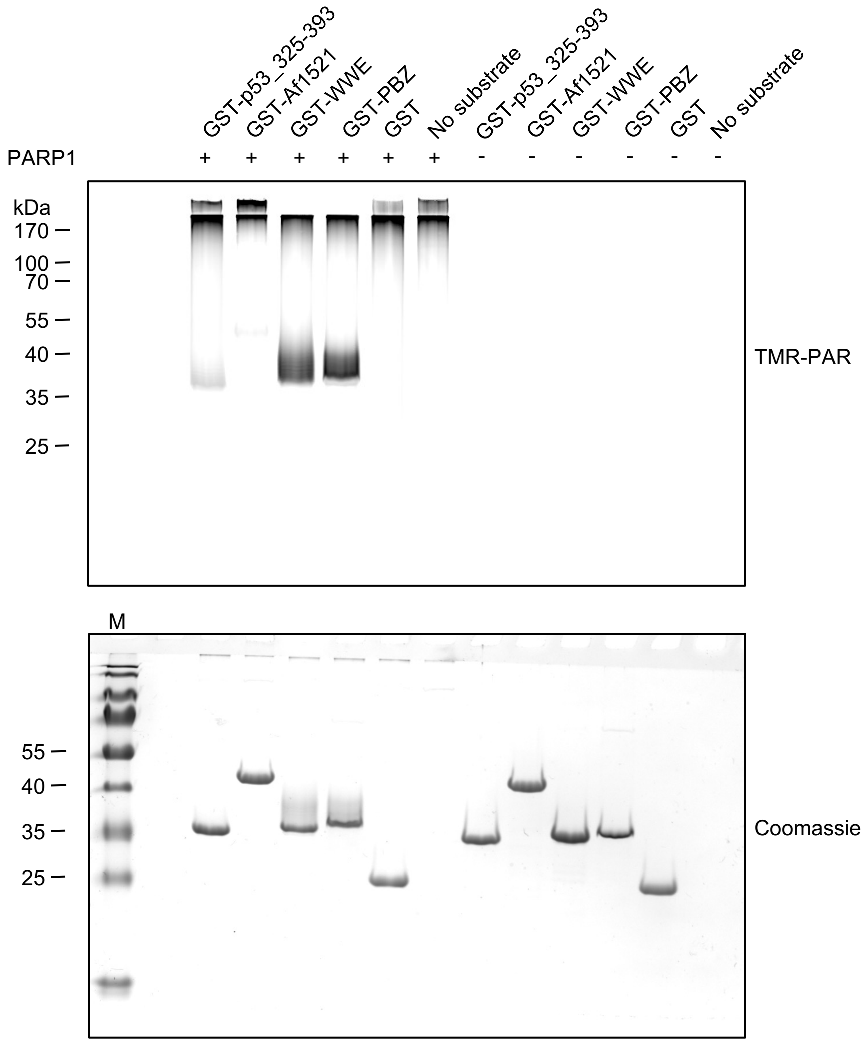

Figure S6. Uncropped gel images from Figure 3A. M: Molecular weight marker

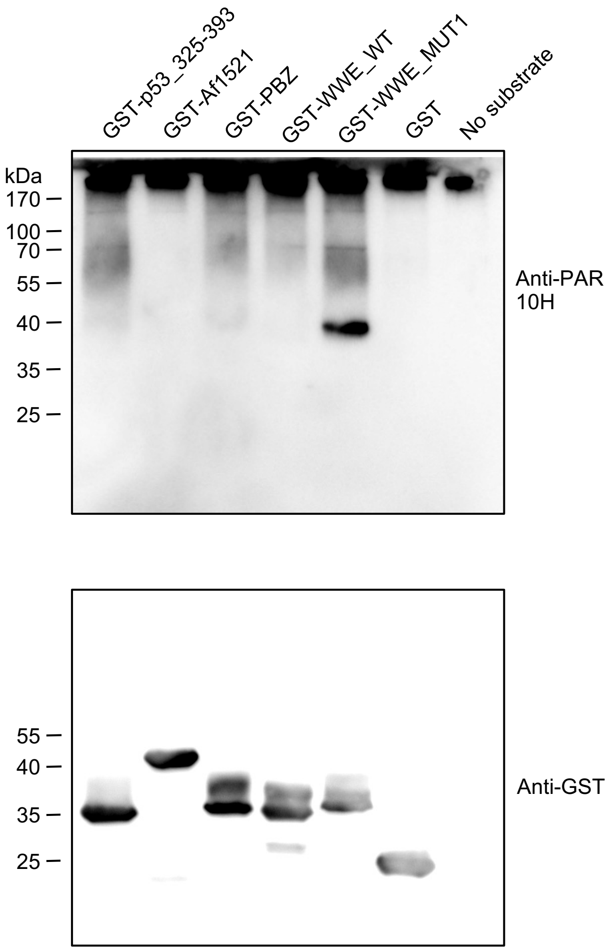

Figure S7. Uncropped Western blots from Figure 3C.

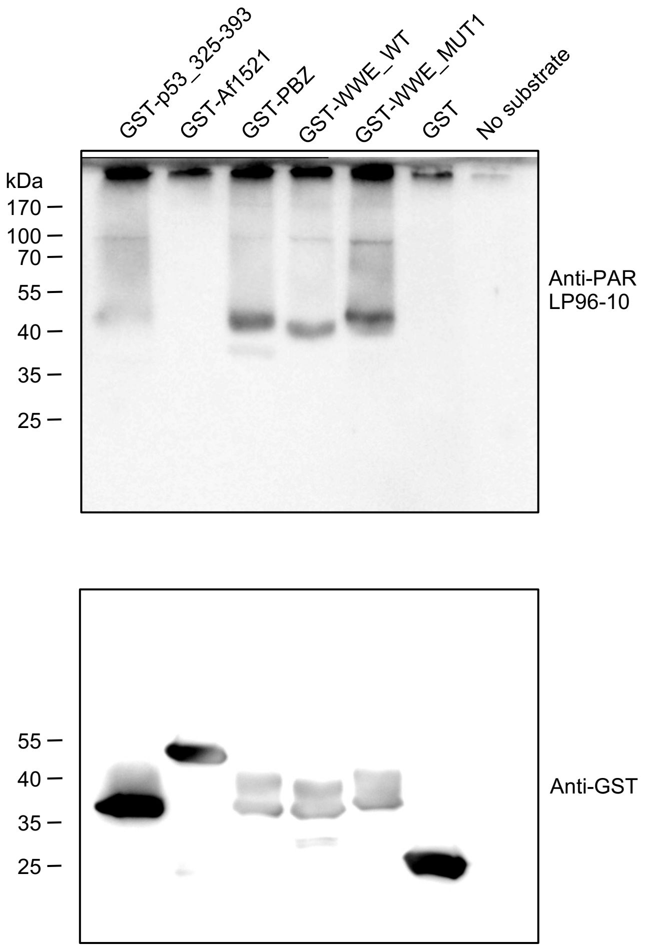

Figure S8. Uncropped Western blots from Figure 3D.

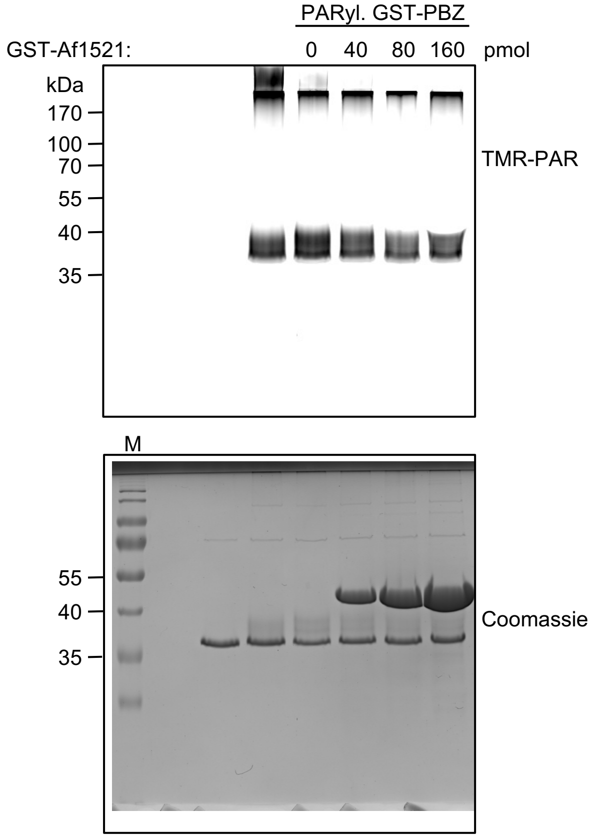

Figure S9. Uncropped gel images from Figure 3E. M: Molecular weight marker

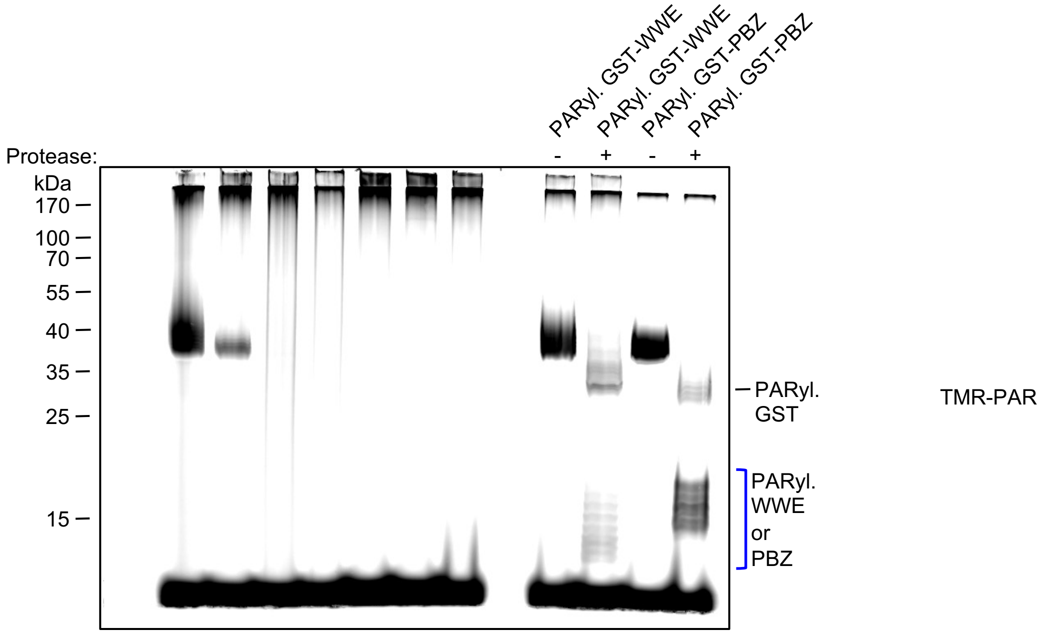

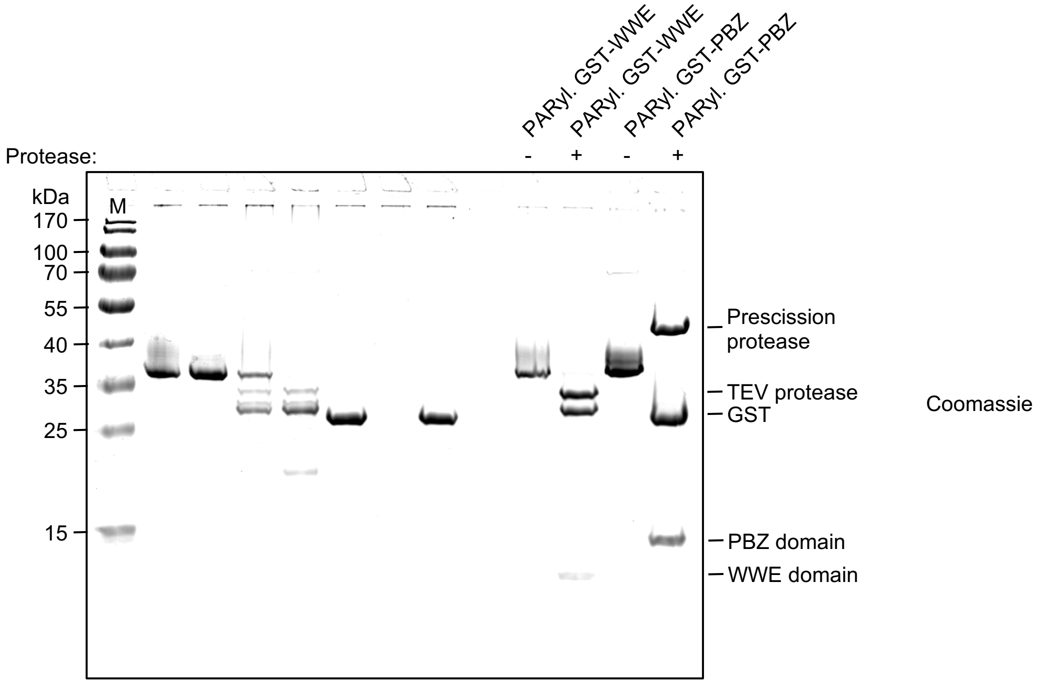

Figure S10. Uncropped gel images from Figure 3F. M: Molecular weight marker

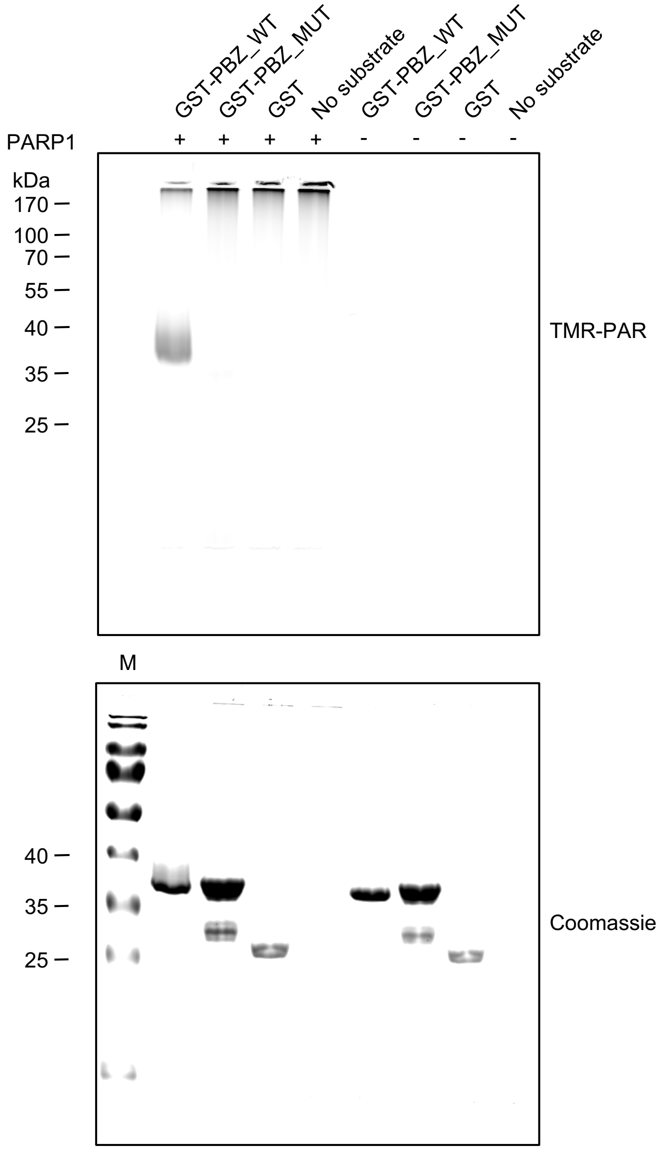

Figure S11. Uncropped gel images from Figure 4A. M: Molecular weight marker

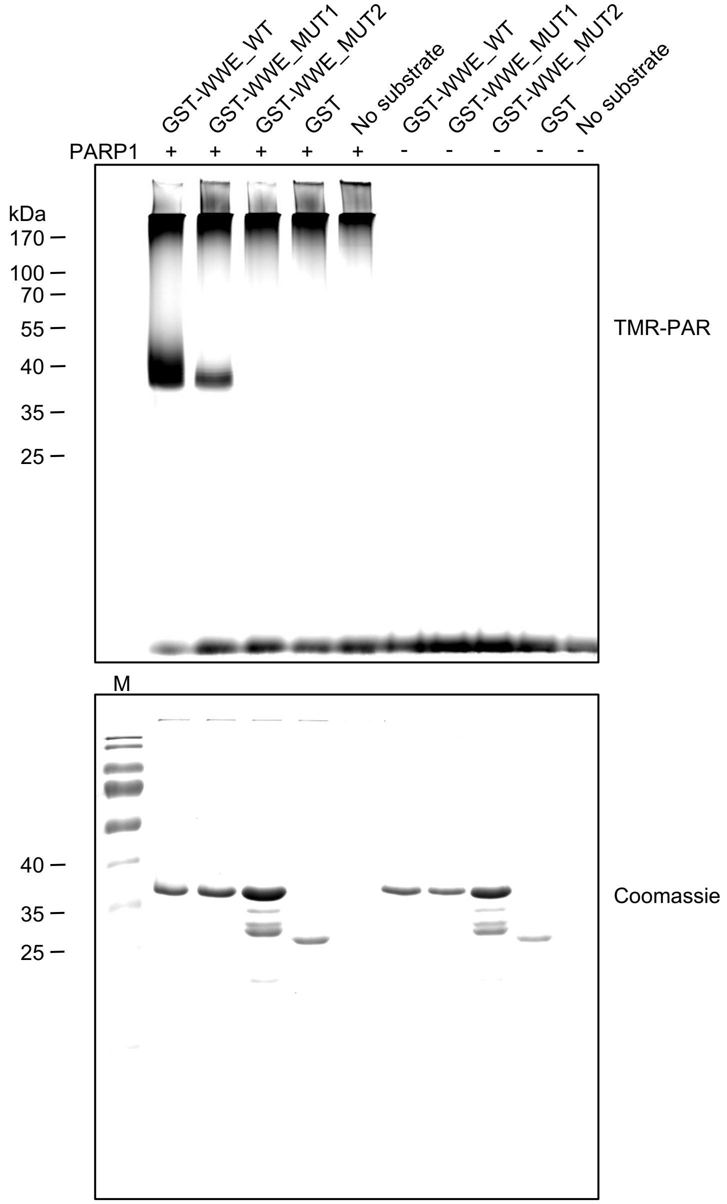

Figure S12. Uncropped gel images from Figure 4B. M: Molecular weight marker

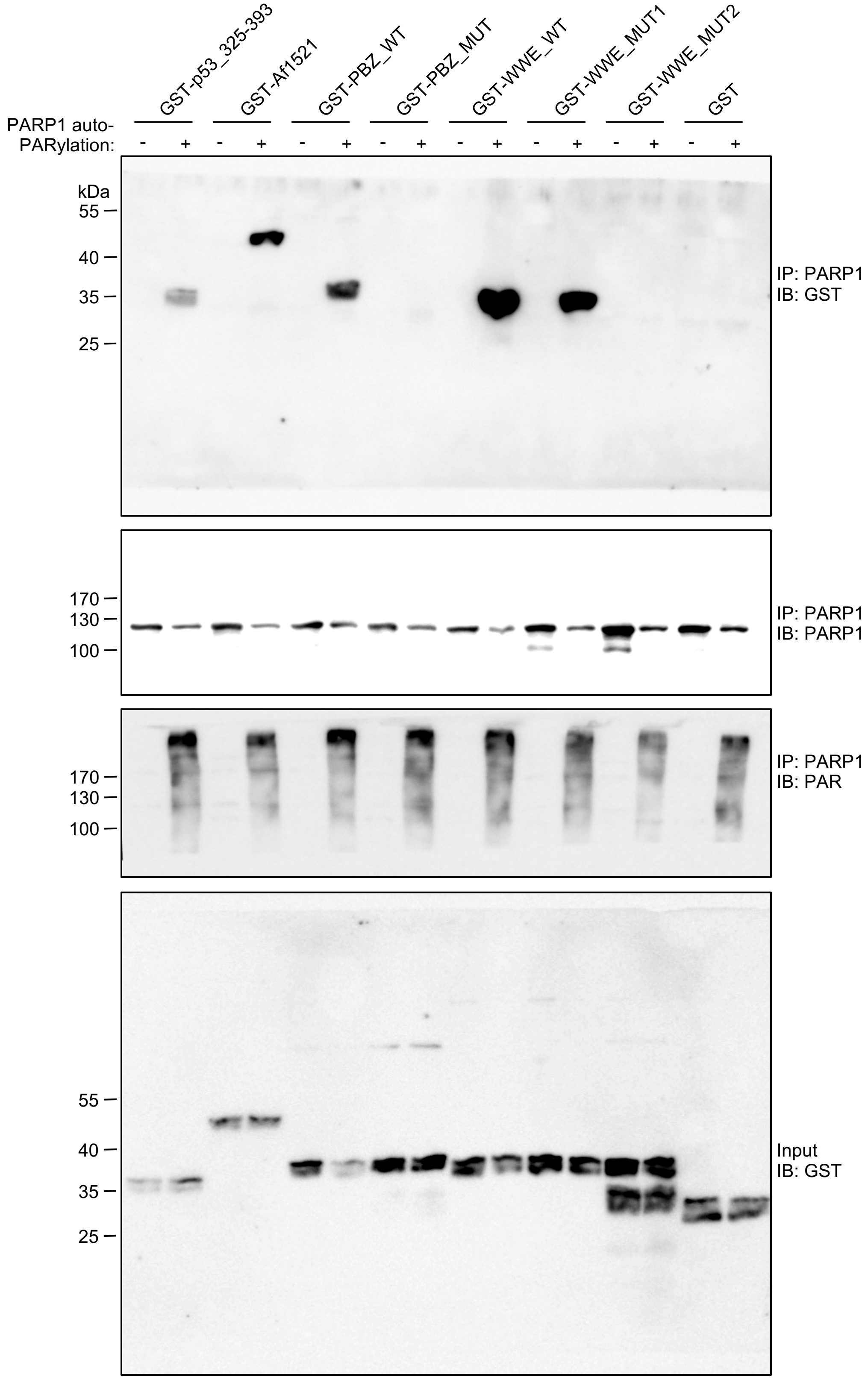

Figure S13. Uncropped Western Blot images from Figure 4E.

Table S1

Name	Sequence (5' to 3')
Oligo_01	ACTTCCAATCCTACGGGGATGATCGTCGTGGTGGCAG
Oligo_02	ATACGGCCTCTCCCTGCGATCCTGTCTGTGC
Oligo_03	ACAGGTTCTCTGCCTGTGCATCTTTCAGTTCCAGT
Oligo_04	TAAGGATCCGGCTGCTAACAAAGCCCGAAAGG
Oligo_05	GCTGGATTTCTGGCTGTTGCTGATCTTG
Oligo_06	CAAGATCAGCAACAGCCAGAAATCCAGC
Oligo_07	CATGGACGTCGCGCGAAGATTAAGCG
Oligo_08	CGCTTAATCTTCGCGCGACGTCCATG
Oligo_09	GCCTGAAGCTCCCTATGGACCATCCGCTTATAG
Oligo_10	CTATAAGCGGATGGTCCATAGGGAGCTTCAGGC
Oligo_11	GACATCCGCCATGTATGGGGCAAACGCCTATAGG
Oligo_12	CCTATAGGCGTTTGCCCCATACATGGCGGATGTC
